# Structures of human PKG reveal cGMP-selectived activation mechanisms

**DOI:** 10.1186/2050-6511-14-S1-O16

**Published:** 2013-08-29

**Authors:** Gilbert Y Huang, Jeong J Kim, Albert S Reger, Robin Lorenz, Eui-Whan Moon, Chi Zhao, Darren E Casteel, Daniela Bertinetti, Bryan VanSchouwen, Rajeevan Selvaratnam, James W Pflugrath, Banumathi Sankaran, Giuseppe Melacini, Friedrich W Herberg, Choel Kim

**Affiliations:** 1 Verna and Marrs McLean Department of Biochemistry and Molecular Biology, Baylor College of Medicine, Houston, TX 77030, USA; 2 Department of Pharmacology, Baylor College of Medicine, Houston, TX 77030, USA; 3 Department of Biochemistry, University of Kassel, Kassel, Germany; 4 Department of Chemistry, Rice University, Houston, TX 77005 USA; 5 Department of Medicine, University of California, San Diego, La Jolla, CA 92093, USA; 6 Department of Chemistry and Chemical Biology, McMaster University, Hamilton, Ontario, Canada; 7 Rigaku Americas, The Woodlands, TX 77381, USA; 8 Berkeley Center for Structural Biology, Lawrence Berkeley National Laboratory, 1 Cyclotron Road, Berkeley, CA, USA

## Background

Cyclic guanosine monophosphate (cGMP) is a key secondary messenger that is produced in response to nitric oxide. One of the key mediators of cGMP signaling, cGMP-dependent protein kinase (PKG), is activated upon binding to cGMP and phosphorylates downstream substrates in a process required for important physiological processes such as vasodilation, nociception, and memory formation. PKGs are also known to mediate most effects of drugs that increase cellular cGMP levels, including nitric oxide-releasing agents and phosphodiesterase inhibitors, which are used for the treatment of angina pectoris and erectile dysfunction, respectively. It is known that PKG is preferentially activated by cGMP over cAMP roughly 60-100 fold – however, the molecular mechanism by which cGMP is distinguished from a structurally similar messenger, cAMP, is poorly defined. Using competition fluorescence polarization (FP), X-ray crystallography, and in vitro kinase assays, we sought to understand the molecular basis for cGMP selectivity in PKGI.

## Results

We determined using competition FP that the C-terminal cGMP-binding domain (CNB-B) is a minimal construct that has 200-fold selectivity for cGMP. Using X-ray crystallography, we solved the structures of CNB-B bound to cGMP (1.65 Å) and in the apo form (2.0Å). The CNB-B:cGMP complex structure reveals that highly conserved residues om strand beta 5 and the C-helix of PKGIβ interact specifically with the guanine moiety through hydrogen bonding and π stacking interactions, providing over 240-folds cGMP selectivity. Mutagenesis of these residues demonstrates their importance not only in cGMP selectivity, but also in activation. Surprisingly, comparison with the apo structure reveals that the pocket is not preformed, but assembled through major structural rearrangements of the helical domain.

Our affinity measurements demonstrate that CNB-B is a minimal domain that provides cGMP selectivity for PKG I, while our X-ray structures of CNB-B reveal contacts unique to PKG that confer cGMP selectivity. Furthermore, kinase assays show that these contacts play a role in cGMP-dependent activation of the full-length protein.

## Conclusion

Taken together, our data suggest that PKGI has a unique mode of recognition for cGMP and describes conformational changes required for activation of the full-length kinase.

